# Test-retest reliability of handgrip muscle function among Australian rules football athletes

**DOI:** 10.1016/j.jesf.2026.200476

**Published:** 2026-04-21

**Authors:** Liam O. Mathews, Hazel R. Jaekel, Nicola D. Ridgers, Mark J. Catley, Grant R. Tomkinson

**Affiliations:** a Alliance for Research in Exercise, Nutrition and Activity (ARENA), School of Allied Health and Human Performance, Adelaide University, Adelaide, SA, Australia; b Innovation, IMPlementation and Clinical Translation (IIMPACT), School of Allied Health and Human Performance, Adelaide University, Adelaide, SA, Australia

**Keywords:** Athletic performance, Australian football, Hand strength, Reproducibility of results

## Abstract

**Background/objective:**

Advancements in handgrip dynamometry provide unique opportunities to measure multiple aspects of handgrip muscle function. The aim of this study was to quantify the test-retest reliability of handgrip muscle function among athletes using digital handgrip dynamometry.

**Methods:**

Eighty-five Australian football athletes (aged 18–36 years) competing in men's and women's national and state league competitions were included. Handgrip muscle function (i.e., handgrip strength [HGS], handgrip rate of force development [RFD]) was assessed using digital handgrip dynamometry. Reliability was quantified as the systematic error (using the difference in means), random error (using the typical error), and test-retest correlations (using the intraclass correlation coefficient [ICC]).

**Results:**

Participants were predominantly males (*n* = 52 [61%]) and competed in national competitions (*n* = 54 [64%]). For HGS, the standardised systematic error was negligible (mean difference [95% CI]: 0.04 [-0.02, 0.10]), the standardised random error was small (typical error [95% CI]: 0.19 [0.17, 0.22]), and the test-retest correlation was nearly perfect (ICC_3,1_ [95% CI]: 0.96 [0.94, 0.98]). For handgrip RFD, the standardised systematic error was small (mean difference [95% CI]: 0.28 [0.15, 0.41]), the standardised random error was moderate (typical error [95% CI]: 0.44 [0.38, 0.52]), and the test-retest correlation was very high (ICC_3,1_ [95% CI]: 0.84 [0.76, 0.89]).

**Conclusions:**

Digital handgrip dynamometry may be used to measure HGS and handgrip RFD with very high to nearly perfect reliability. Practitioners could consider using digital handgrip dynamometry to assess athletes, with greater confidence for profiling or monitoring changes in HGS compared with handgrip RFD.

## 1 Introduction

Handgrip strength (HGS), a maximal isometric grip force task assessed using handgrip dynamometry, is a feasible, safe, valid, and reliable measure of strength capacity.^1^, ^2^, ^3^ Although strength capacity cannot be defined by a single measure, HGS has moderate-to-high convergent validity when compared to more complicated assessments of whole-body and major muscle group (knee extensor) strength (*r* = 0.74–0.89).^3^^,^^4^ This strength capacity assessment is becoming more affordable with evidence of very high agreement between low cost and traditional dynamometers.^5^ HGS is commonly assessed in sport settings, with higher HGS associated with better athletic performance in contact sports requiring substantial hand-gripping and force application like American football (Gridiron), rugby, judo, and wrestling.^6^^,^^7^ It is for these reasons why HGS appears suitable as a general strength capacity measure among contact sport athletes.

However, HGS is an incomplete measure of muscle function because it only assesses strength capacity. Traditional HGS protocols have focussed on maximal grip force^8^ and have overlooked other important aspects of muscle function like rate of force development (RFD), which is a known significant, positive correlate of sports and athletic performance.^9^^,^^10^ For example, in sports like Australian football where players need quick, powerful hand movements for marking, tackling, grappling and fast ball control, handgrip RFD may be as important—or more important—than HGS. Recent technological advancements have led to the development of digital handgrip dynamometers, which provide unique opportunities to measure additional functional aspects conveniently and simultaneously with HGS. This may be important in sport settings because functional assessments can be used to profile and monitor athletes as well as guide resistance exercise prescriptions.^11^, ^12^, ^13^ Despite its potential significance within sport settings, little research has examined the reliability of both HGS and handgrip RFD using digital handgrip dynamometry.

Test-retest reliability (herein termed *reliability*) is the degree to which a test produces the same result in the same setting with the same participants on separate occasions.^14^ Reliability is important when assessing an individual with single or repeated measurements (i.e., measuring weekly, monthly, or annual changes), estimating the magnitude of individual responses to intervention, comparing different tests or assessors, and estimating sample sizes in experimental studies.^14^ While the reliability of HGS using traditional dynamometry has been well documented among general and athlete populations,^2^^,^^6^ data on the reliability of multiple functional aspects using digital handgrip dynamometry are scant. Recently, Maurya et al.^15^ examined the reliability of digital handgrip muscle function in young women, finding very high (i.e., intraclass correlation coefficient [ICC] = 0.70–0.89) to nearly perfect (i.e., ICC ≥0.90) test-retest correlations for HGS and handgrip RFD (ICC_2,1_: 0.89–0.92). No study to our knowledge has quantified the reliability of digital handgrip muscle function among athletes. Quantifying the reliability of digital handgrip dynamometry in the population of interest (i.e., athletes) is important because reliability statistics are population and measurement system (i.e., setting, equipment, measurer) specific and are, therefore, not transferable.^16^ The aim of this study, therefore, was to quantify the reliability of multiple aspects of handgrip muscle function using digital dynamometry among athletes.

## 2 Methods

### 2.1 Design, participants and settings

Using a repeated measures design, handgrip muscle function assessments were conducted at the same time of day at two club training sessions spaced approximately 7 days apart. Australian football athletes from the men's and women's senior squads competing in national (Australian Football League [AFL] and Australian Football League Women's [AFLW]) or state (South Australian National Football League [SANFL] and South Australian National Football League Women's [SANFLW]) football competitions were recruited. Australian football is a contact sport played on a large oval-shaped grass field that requires various high-intensity dynamic movements such as sprinting, jumping, marking and tackling.^17^ As Australian football performance is influenced by multiple aspects of muscle function, its athletes represent a suitable athletic group to sample because they possess a broad range of physical and functional characteristics to meet their sport demands.

The target sample size was 50 adult participants (aged 18+ years), which according to Hopkins^14^ provides adequate precision for the typical error (i.e., the standard measure of reliability). Participation was voluntary, and athletes gave written informed consent after being told the study aims. Participants were ineligible if they self-reported as: (a) currently injured, (b) having an underlying condition (e.g., carpal tunnel syndrome), for which handgrip muscle function testing would be contraindicated, or (c) having had a major hand/wrist injury (e.g., dislocation, partial/complete fracture) within the past 3 months. Assessments occurred within a single competitive season between August 2023 and February 2024 during regular training, either as part of normal athlete rotations or in short blocks (∼45 min) before or after sessions, depending on club schedules. While this sample is larger than the target sample size, it was unreasonable to exclude team members because testing was performed in club settings. Participants were medically cleared to participate by their clubs. Institutional Human Research Ethics Committee approval was received.

### 2.2 Measures

Handgrip muscle function was objectively assessed using a factory-calibrated digital handgrip dynamometer (DynaMo 1.3.8, VALD, Australia) and the modified American Society of Hand Therapists protocol.^18^ Testing was conducted by trained assessors who explained and showed how to do the procedures before assessment. The protocol required participants to squeeze the dynamometer while seated upright, with their shoulder adducted against their torso, elbow flexed to 90°, and their forearm and wrist in neutral (between 0 and 30° wrist extension).^17^ The dynamometer was fitted to the participant's hand size, with the distance between the handles recorded and standardised for retesting. Once fitted, a submaximal practice trial was allowed to verify the participant's interpretation of the testing procedures and to check the dynamometer was functional. Starting with their right hand, participants were instructed to “squeeze the handle as hard and as fast as possible” to produce a rapid and maximal effort, and were verbally encouraged (“Go, Go, Go”) during task performance. Participants alternated between hands with a standardised 15 s rest between trials, resulting in a minimum of 30 s rest between efforts from the same hand.^19^ Three trials per hand were always performed. HGS was reported as the peak force measured in kilograms (kg) with handgrip RFD measured as the peak instantaneous RFD smoothed with a 100-ms window in kilograms per second (kg/s). To align with standardised protocols, the maximum value irrespective of hand was used in statistical analyses.^20^

Prior to testing, participants had their stature and body mass measured in athletic training clothing (e.g., shorts and t-shirt) and without shoes. Stature was measured using a stadiometer to the nearest 0.1-cm (Seca 213 Portable Stadiometer, Hamburg, Germany). Body mass was measured using weighing scales to nearest 0.1-kg (Tanita UM-051 weighing scale, Tokyo, Japan). At the time of consent, participants also self-reported demographic information (e.g., age, sex, competitive standard, years played at current competitive standard) using an online survey (Qualtrics XM, Provo, Utah, USA).

### 2.3 Statistical methods

Pooled descriptive characteristics of participants were reported as frequencies (percentage) for categorical variables and means (standard deviation [SD]) for continuous variables. Bland-Altman absolute difference plots were visually examined where the mean test-retest values (*x*-axis) were plotted against the absolute test-retest differences (i.e., the bias) (*y*-axis). Non-uniformity of errors was examined by inspecting mean-difference plots and by calculating Pearson's correlations between the absolute differences and the means of the duplicate measurements. Because there was evidence of slight proportional error, data were log transformed prior to reliability analyses and then back-transformed to provide interpretable relative (percentage) error statistics, as recommended by Hopkins.^14^ Reliability was quantified as the: (a) systematic (bias) error, (b) random (within-subject) error, and (c) test-retest correlation. Systematic errors were expressed as absolute, percent, and standardised differences in means. Absolute difference in means was calculated as the test mean minus the retest mean; percent differences as the absolute difference in means divided by the test-retest mean multiplied by 100; and standardised differences as the absolute difference in means divided by the pooled between-subjects standard deviation. Positive systematic errors indicated larger test measurements and negative systematic errors indicated smaller test measurements. Standardised effect size (ES) for systematic error was interpreted as negligible (ES < 0.20), small (ES = 0.20–0.59), moderate (ES = 0.60–1.19), large (ES = 1.20–1.99), very large (ES = 2.00–3.99), and extremely large (ES ≥ 4.00).^21^ Random error was expressed as the typical error, calculated as the standard deviation of the differences divided by $\sqrt{2}$ and expressed as absolute, percent (relative to the mean test-retest values), and standardised (relative to the pooled between-subjects standard deviation) typical errors. Standardised ES for random error was interpreted as negligible (ES < 0.10), small (ES = 0.10–0.29), moderate (ES = 0.30–0.59), large (ES = 0.60−0.99), very large (ES = 1.00−1.99), and extremely large (ES ≥ 2.00).^22^ Test-retest correlation was examined by the two-way mixed effects ICC_3,1_ and interpreted as negligible (ICC <0.10), small (ICC = 0.10–0.29), moderate (ICC = 0.30–0.49), high (ICC = 0.50–0.69), very high (ICC = 0.70–0.89), and nearly perfect (ICC ≥0.90).^21^ Reliability was visualised as Bland-Altman percentage difference plots. Finally, because different reporting protocols are used for HGS and handgrip RFD,^23^ we also assessed the reliability of these protocols using test-retest correlations as a sensitivity analysis.

## 3 Results

Eighty-five participants consented, met eligibility criteria, and provided test-retest data. Descriptive statistics for included participants are shown in Table 1. Participants were predominantly males (*n* = 52 [61%]), competed in national competitions (*n* = 54 [64%]), and played on-ball, wing, or non-key back/forward positions (termed *nomadic* positions; *n* = 57 [67%]). Male and female players were similar in age and years played at their current competitive standard.

**Table 1:** Descriptive characteristics of Australian football athletes (mean [SD]).

Measurement	Female (*n* = 33)	Male (*n* = 52)	Overall (*n* = 85)
Age (years)	23 (4)	23 (3)	23 (4)
Height (cm)	170 (6)	187 (7)	180 (11)
Experience (years)	4 (2)	5 (3)	5 (3)
Mass (kg)	69 (8)	88 (9)	81 (13)
HGS (kg)	40 (6)	62 (9)	53 (13)
Handgrip RFD (kg/s)	224 (62)	323 (90)	272 (92)

Abbreviations: cm = centimetres; HGS = handgrip strength; kg = kilograms; kg/s = kilograms per second; RFD = rate of force development; SD = standard deviation.

Table 2 shows the results of the reliability analysis for HGS and handgrip RFD. For HGS, the standardised systematic error was negligible (mean difference [95% CI]: 0.04 [-0.02, 0.10]), the standardised random error was small (typical error [95% CI]: 0.19 [0.17, 0.22]), and the test-retest correlation was nearly perfect (ICC_3,1_ [95% CI]: 0.96 [0.94, 0.98]). For handgrip RFD, the standardised systematic error was small (mean difference [95% CI]: 0.28 [0.15, 0.41]), the standardised random error was moderate (typical error [95% CI]: 0.44 [0.38, 0.52]), and the test-retest correlation was very high (ICC_3,1_ [95% CI]: 0.84 [0.76, 0.89]). Our sensitivity analysis showed negligible differences in test-retest correlations across reporting protocols (Supplementary Table 1).

**Table 2:** Results of the reliability analysis for Australian football athletes (*n* = 85).

Muscle function measure	Test Mean (SD)	Retest Mean (SD)	Systematic error			Random error			ICC_3,1_ (95%CI)
			Absolute difference (95%CI)	Percent difference (95%CI)	ES difference (95%CI)	Absolute TE (95%CI)	Percent TE (95%CI)	ES TE (95%CI)	
HGS (kg)	54 (13)	53 (13)	0.4 (-0.4, 1.2)	1.1 (-0.5, 2.6)	0.04 (-0.02, 0.10)	2.6 (2.2, 3.0)	5.4 (4.8, 6.6)	0.19 (0.17, 0.22)	0.96 (0.94, 0.98)
Handgrip RFD (kg/s)	285 (97)	259 (90)	25.9 (14.7, 37.2)	9.7 (4.3, 13.2)	0.28 (0.15, 0.41)	36.8 (32.0, 43.3)	16.1 (15.0, 20.9)	0.44 (0.38, 0.52)	0.84 (0.76, 0.89)

Notes: HGS and handgrip RFD values are reported in kg and kg/s, respectively, for the test mean (SD), retest mean (SD), absolute difference (95%CI), and absolute TE (95%CI). The test (SD) and retest mean (SD) were calculated from raw data. Positive systematic errors indicated larger test measurements and negative systematic errors indicated smaller test measurements. Standardised ES for systematic error was interpreted as negligible (ES < 0.20), small (ES = 0.20–0.59), moderate (ES = 0.60–1.19), large (ES = 1.20–1.99), very large (ES = 2.00–3.99), and extremely large (ES ≥ 4.00).^21^ Standardised ES for random error was interpreted as negligible (ES < 0.10), small (ES = 0.10–0.29), moderate (ES = 0.30–0.59), large (ES d = 0.60−0.99), very large (ES = 1.00−1.99), and extremely large (ES ≥ 2.00).^22^ Test-retest correlations were interpreted as negligible (ICC <0.10), small (ICC = 0.10–0.29), moderate (ICC = 0.30–0.49), high (ICC = 0.50–0.69), very high (ICC = 0.70–0.89), and nearly perfect (ICC ≥0.90).^21^.

Abbreviations: 95% CI = 95 percent confidence interval; ES = standardised effect size; HGS = handgrip strength; ICC_3,1_ = intra-class correlation coefficient, model 3,1; kg = kilogram; kg/s = kilogram per second; RFD = rate of force development; SD = standard deviation; TE = typical error.

**Fig. 1:**
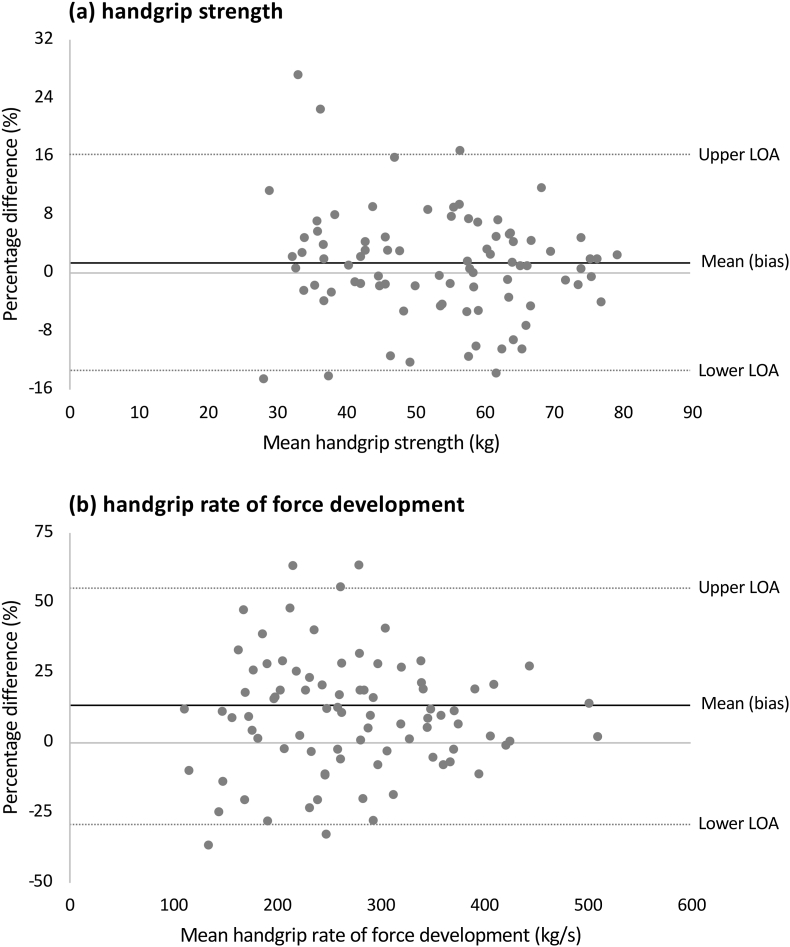
Bland-Altman percentage difference plots for (a) handgrip strength and (b) handgrip rate of force development among Australian football athletes. Abbreviations: LOA = limit of agreement; kg = kilograms; kg/s = kilograms per second; % = percent.

Fig. 1 shows the Bland-Altman percentage difference plots for HGS and handgrip RFD. The systematic errors were 1.1% and 9.7% for HGS and RFD, respectively. The 95% limits of agreement (LOA) were -13.9% to 16.1% for HGS and -34.9% to 54.3% to for RFD.

## 4 Discussion

This study aimed to quantify the test-retest reliability of handgrip muscle function among athletes using digital handgrip dynamometry. Overall, digital handgrip dynamometry demonstrated very good reliability for both HGS and handgrip RFD, evidenced by negligible to small systematic errors, small to moderate random errors, and very high to nearly perfect test-retest correlations. However, the reliability of handgrip RFD was somewhat worse compared to HGS with larger random and systematic errors and a weaker test-retest correlation. These findings have important implications for handgrip muscle function testing among contact sport athletes.

Our finding of a nearly perfect test-retest correlation for HGS is consistent with that of a recent systematic review on the reliability of field-based fitness tests among adults, which included HGS.^2^ Using test-retest HGS reliability data from 22 studies on apparently healthy adults aged 18–64 years, nearly perfect test-retest correlations were reported for a range of digital and analogue handgrip dynamometers.^2^ While the studies reviewed by Cuenca Garcia et al.^2^ comprised mainly adults from the general population, similar reliability results for HGS have been found for athletes^24^ and lower-body assessments such as the squat jump, counter-movement jump, and isometric mid-thigh pull.^11^^,^^24^, ^25^, ^26^, ^27^ Collectively, these findings indicate that the reliability of HGS using digital dynamometry among Australian football athletes compares favourably to those reported in general and athletic populations and for other common strength assessments.

While incorporating RFD assessments into digital handgrip dynamometers is new, the reliability of lower-body RFD has been more widely examined. Hernández-Davó and Sabido^28^ reviewed the reliability of RFD across 20 studies that predominantly used lower-body assessments among healthy adults aged 18–50 years, finding that RFD assessments generally demonstrated very high to nearly perfect reliability. While none of the reliability studies reviewed by Hernández-Davó and Sabido^28^ assessed handgrip RFD, some studies have quantified the reliability of handgrip RFD using digital handgrip dynamometry among young adults.^15^^,^^29^ Our finding of very high test-retest correlation for handgrip RFD is consistent with that reported by Demura et al.^29^ yet is somewhat lower than that reported by Maurya et al.^15^ Other studies have reported lower reliability statistics for RFD than for strength assessments in both general population and athletes.^23^^,^^28^^,^^30^^,^^31^ These findings contrast those of Maurya et al.^15^ who found that both HGS and handgrip RFD measures had nearly perfect test-retest correlations. The contrasting findings between the present study and those of Maurya et al.^15^ may be due to differences in dynamometers, dynamometer sampling rates, testing protocols, and sample demographics. Despite this, the reliability of handgrip RFD among Australian football athletes compares well with that seen in general and athletic populations and for other common RFD assessments. This suggests that digital handgrip dynamometers may be used among athletic and general populations to assess handgrip RFD as they demonstrate comparable reliability to lower body RFD assessments, which typically require more expensive and bulkier equipment.

It is important to note that although test-retest correlations summarize the total error, they do not separate the systematic and random errors.^14^ Correlations are also influenced by sample heterogeneity, whereas random errors are not. As such, the random error is considered a key indicator for determining whether an assessment has ‘acceptable’ reliability. Our finding of low random error in digital HGS is consistent with other strength measures across a variety of assessments,^11^^,^^15^^,^^24^ and suggests that the reliability of HGS is comparable to other strength assessments. In contrast, handgrip RFD demonstrated moderate random error, which was more than double that observed for HGS, and small systematic error. While repeat assessments were conducted in a field-setting rather than in a controlled laboratory setting, it appears that the pre-test activity exposures had more of an influence on the measurement error observed for handgrip RFD than for HGS. This may be because RFD is highly sensitive to neuromuscular readiness and fatigue.^32^ As a result, the lack of standardised pre-testing conditions likely increased within-participant biological error between sessions, affecting handgrip RFD reliability more than HGS reliability. Future studies should examine the impact of the testing setting and pre-test activity exposure on measurement reliability.

Another application of reliability data is the ability to assess real (error-free) changes within individuals. According to Hopkins,^14^ a test result must change by more than 1.5 or 2.0 times the typical error (corresponding to about 70% or 85% certainty) to be considered error-free (i.e., exceeding measurement error). For example, with percent typical errors of 5.4% for HGS and 16.1% for handgrip RFD, HGS would need to increase from 53 kg to more than 57.3 kg or 58.7 kg (8.1% or 10.8% change), and handgrip RFD from 272 kg/s to more than 338 kg/s or 360 kg/s (24.2% or 32.2% change). Although defining 'acceptable' change is challenging for athlete monitoring, our reliability data suggest that digital handgrip dynamometry can detect typical resistance training improvements in HGS, but not handgrip RFD, in contact sport athletes.^33^^,^^34^ To improve reliability and to better detect functional changes in athletes, strategies such as standardising pre-test activity exposure (e.g., within the previous 24 h) and reducing diurnal variability (e.g., testing at the same time of day and week) are recommended.

The strengths of this study include the sufficiently powered sample, consistent testing procedures using the same equipment, and the inclusion of both male and female Australian football athletes from two competitive standards, making these findings more applicable to elite and sub-elite athletes of both sexes. However, a key limitation was the inability to standardise pre-test activity exposure between testing and retesting due to assessments being conducted during regular club trainings. Although efforts were made to standardise testing conditions (e.g., time of testing, assessment protocol), the athletes’ training environments and requirements impacted our ability to standardise pre-testing conditions. However, this can be seen as a strength, improving the ecological validity of our reliability estimates. This is because such conditions match real-world sports club environments where variations in match schedules, travel, training loads, and player fatigue naturally influence testing.

## 5 Conclusion

This study found that digital handgrip dynamometry demonstrated very high to nearly perfect reliability overall for HGS and handgrip RFD among contact sports athletes. Strategies like standardising pre-test activity exposure and minimising diurnal variability are needed to improve measurement reliability, especially for handgrip RFD which demonstrated larger random and systematic errors compared to HGS. This exploratory study is the first to assess the reliability of HGS and handgrip RFD among contact sport athletes in Australian footballers. Practitioners may consider using digital handgrip dynamometry to assess multiple functional capabilities among athletes, with greater confidence for profiling or monitoring changes in HGS compared with handgrip RFD.

## Data statement

The datasets analysed in this review are available from the corresponding author on reasonable request.

## CRediT authorship contribution statement

Liam Mathews, Mark Catley, Nicola Ridgers and Grant Tomkinson conceived the idea for the cross-sectional study. Liam Mathews and Hazel Jaekel contributed to the data collection. Mark Catley, Nicola Ridgers and Grant Tomkinson contributed to the supervision of the study. Liam Mathews and Grant Tomkinson contributed to the data analysis. Liam Mathews wrote the first draft of the manuscript. All authors provided critical revision of the manuscript and approved the final submission.

## Funding

No financial or material support of any kind was received for the work described in this article.

## Declaration of interest

Grant Tomkinson is a member of the Editorial Board (Associate Editor) of Journal of Exercise Science & Fitness. He was not involved in the journal's peer review process of, or decisions related to, this manuscript. The author(s) declare no other conflicts of interest relevant to this article.
