# Supplementary material for: Test-retest reliability of handgrip muscle function among Australian rules football athletes
Source: J Exerc Sci Fit. 2026 Apr 21;24(3):200476. doi: 10.1016/j.jesf.2026.200476 (PMC13127334; doi:10.1016/j.jesf.2026.200476)
Supplement: Multimedia component 1 [file mmc1.docx]

**Supplementary Table 1.** Test-retest correlations across reporting protocols for Australian football athletes (*n* = 85).

|  | **ICC**_3,1_ **(95%CI)** | | |
| --- | --- | --- | --- |
| **Muscle function measure** | **Maximum (left)** | **Maximum (right)** | **Average of maxima** |
| HGS | 0.96 (0.93, 0.97) | 0.96 (0.94, 0.98) | 0.97 (0.95, 0.98) |
| Handgrip RFD | 0.81 (0.72, 0.87) | 0.77 (0.66, 0.84) | 0.86 (0.79, 0.90) |

Notes: Test-retest correlations were interpreted as negligible (ICC < 0.10), small (ICC = 0.10–0.29), moderate (ICC = 0.30–0.49), high (ICC = 0.50–0.69), very high (ICC = 0.70–0.89), and nearly perfect (ICC ≥ 0.90).^21^

Abbreviations: 95% CI = 95 percent confidence interval; HGS = handgrip strength; ICC_3,1_ = intra-class correlation coefficient, model 3,1; RFD = rate of force development.
